# Automatic differentiation of Glaucoma visual field from non-glaucoma visual filed using deep convolutional neural network

**DOI:** 10.1186/s12880-018-0273-5

**Published:** 2018-10-04

**Authors:** Fei Li, Zhe Wang, Guoxiang Qu, Diping Song, Ye Yuan, Yang Xu, Kai Gao, Guangwei Luo, Zegu Xiao, Dennis S. C. Lam, Hua Zhong, Yu Qiao, Xiulan Zhang

**Affiliations:** 1 Zhongshan Ophthalmic Center, State Key Laboratory of Ophthalmology, Sun Yat-sen University, Guangzhou, China; 2 Guangdong key lab of Computer Vision & Virtual Reality, Multimedia Research Center, Shenzhen Institutes of Advanced Technology, Chinese Academy of Sciences, Shenzhen, China; 3 Department of Ophthalmology, the First Affiliated Hospital of Kunming Medical University, Kunming, China; 4 SenseTime Group Limited, Hong Kong, China; 5 C-MER Dennis Lam Eye Hospital, Shenzhen, China

**Keywords:** Glaucoma, Visual field, Deep learning

## Abstract

**Background:**

To develop a deep neural network able to differentiate glaucoma from non-glaucoma visual fields based on visual filed (VF) test results, we collected VF tests from 3 different ophthalmic centers in mainland China.

**Methods:**

Visual fields obtained by both Humphrey 30–2 and 24–2 tests were collected. Reliability criteria were established as fixation losses less than 2/13, false positive and false negative rates of less than 15%.

**Results:**

We split a total of 4012 PD images from 1352 patients into two sets, 3712 for training and another 300 for validation. There is no significant difference between left to right ratio (*P* = 0.6211), while age (*P* = 0.0022), VFI (*P* = 0.0001), MD (*P* = 0.0039) and PSD (*P* = 0.0001) exhibited obvious statistical differences. On the validation set of 300 VFs, CNN achieves the accuracy of 0.876, while the specificity and sensitivity are 0.826 and 0.932, respectively. For ophthalmologists, the average accuracies are 0.607, 0.585 and 0.626 for resident ophthalmologists, attending ophthalmologists and glaucoma experts, respectively. AGIS and GSS2 achieved accuracy of 0.459 and 0.523 respectively. Three traditional machine learning algorithms, namely support vector machine (SVM), random forest (RF), and k-nearest neighbor (k-NN) were also implemented and evaluated in the experiments, which achieved accuracy of 0.670, 0.644, and 0.591 respectively.

**Conclusions:**

Our algorithm based on CNN has achieved higher accuracy compared to human ophthalmologists and traditional rules (AGIS and GSS2) in differentiation of glaucoma and non-glaucoma VFs.

**Electronic supplementary material:**

The online version of this article (10.1186/s12880-018-0273-5) contains supplementary material, which is available to authorized users.

## Background

Glaucoma is currently the second leading cause of irreversible blindness in the world, [1] which is commonly characterized by sustained or temporary elevation of IOP and defects in visual field. According to population-based studies, prevalence of glaucoma in China was about 3%. [2–4] However, less than 20% glaucoma patients had been diagnosed, especially in rural area. [2] We seriously need better tool and method to assist screening and diagnosis of glaucoma.

Different from other ocular diseases such as corneal diseases or fundus diseases, which can be diagnosed according to obvious anatomical changes, diagnosis of glaucoma depends on the information from various clinical examinations including visual field (VF), optical coherence tomography (OCT) and fundus photo. [1, 5] In clinical practice, VF is widely used as the gold standard to judge whether patients have typical glaucomatous damage. In clinical practice, VF is measured by perimetry. There are several types of perimeters, such as Humphrey Field Analyzer or the Oculus. During VF tests, light spots are flashed at varying intensities at fixed locations in the inner sphere of perimeters. When the subjects see the spots, they should make a response by pressing a button so that doctors would get a report about the light sensitivities at different locations in the subjects’ VF. There are 2 forms of perimetry commonly used in diagnosis and follow-up of glaucoma: 30–2 and 24–2. For ophthalmologists who are not specialized in glaucoma, it may be difficult for them to interpret a single VF report. Specific patterns of defects such as nasal step and arcuate scotoma shown in visual field indicate existence of glaucoma. [6, 7]

Researchers have developed several algorithms based on data from clinical studies, such as Advanced Glaucoma Intervention Study (AGIS) criteria and Glaucoma Staging System (GSS) criteria to grade glaucomatous VFs. [6, 8–10] However, it is hard to diagnose glaucoma depending on VF alone and for early stage glaucoma, even if retinal nerve fiber layer (RNFL) had been damaged there can be no obvious defect in VF. Therefore, it is necessary to develop new algorithm for glaucoma diagnosis.

Machine learning community has developed a family of powerful tools that grant computers the ability to learn from and make predictions on data. Various algorithms have been proposed for solving the classification problem, such as Support Vector Machine, Random Forest, Boosting, etc. However, these algorithms mainly rely on the manually designed features for the task, and may not be able to generalize to unseen data. Recently, deep convolutional neural networks (CNN) have achieved state-of-the-art performance on a variety of tasks in artificial intelligence. It is able to jointly optimize the feature extraction and classification tasks. Deep networks have also been successfully utilized in diagnosis of certain diseases, whose diagnostic process involves mostly imaging reports. Currently machine learning-based automatic diagnosis of diseases depends on input of large amount of clinical data with definite labels. In 2016, machines have been trained to identify diabetic retinopathy (DR) from fundus photography, achieving high sensitivity and specificity. [11] Diagnosis of congenital cataract (CC) by artificial intelligence has also been carried out. [12] With self-adopted deep neural network, machines are able to learn and make accurate diagnosis. Besides DR and CC, researchers also trained neural network to identify preperimetric glaucoma from VF reports. [13]

Unlike DR or CC, diagnosis of glaucoma cannot be simply made upon photos. Thus, we designed this study to investigate the performance of deep neural network to identify glaucomatous VFs from non-glaucomatous VFs and to compare the performance of machine against human ophthalmologists.

## Methods

### Data preparation

The study was approved by the Ethical Review Committee of the Zhongshan Ophthalmic Center and was conducted in accordance with the Declaration of Helsinki for research involving human subjects. The study has been registered in clincaltrials.gov (**NCT: 03268031**). All the visual fields (VFs) were obtained by either Humphrey Field Analyzer 30–2 or 24–2 tests. To guarantee reliability, only VFs with fixation losses of less than 2/13, false positive and false negative rates of less than 15% were selected in the experiments. Representative examples of non-glaucoma and glaucoma PD plots are shown in Additional file 1: Figure S1. The probability map of pattern deviation (PD image) is then cropped from the VF report and resized to 224 × 224 as the input of a deep CNN. All the VFs of both eyes of a single patient are assigned to either training or validation set to avoid data leakage. In this way, we split a total of 4012 PD images into two sets, 3712 for training and another 300 for validation. For data augmentation, we randomly flip the PD images in the training set horizontally to obtain final 7424 training samples. Cross validation is performed by randomly splitting the training and validation sets 3 times and no significant difference is observed. The validation set consists of 150 glaucomatous PD images and 150 non-glaucomatous PD images. The non-glaucomatous PD images include 50 images with only cataract and 150 images with no ocular disease, retinal diseases or neuro-ophthalmic diseases.

### Diagnostic criteria of Glaucoma

Glaucoma was diagnosed with similar criteria to UKGTS study. [14] VFs of patients who have glaucomatous damage to optic nerve head (ONH) and reproducible glaucomatous VF defects were included. A glaucomatous VF defect was defined as a reproducible reduction of sensitivity compared to the normative database in reliable tests at: (1) two or more contiguous locations with *P* < 0.01 loss or more, (2) three or more contiguous locations with *P* < 0.05 loss or more. ONH damage was defined as C/D ratio ≥ 0.7, thinning of RNFL or both, without a retinal or neurological cause of VF loss.

### Deep CNN for VF differentiation

**Fig. 1:**
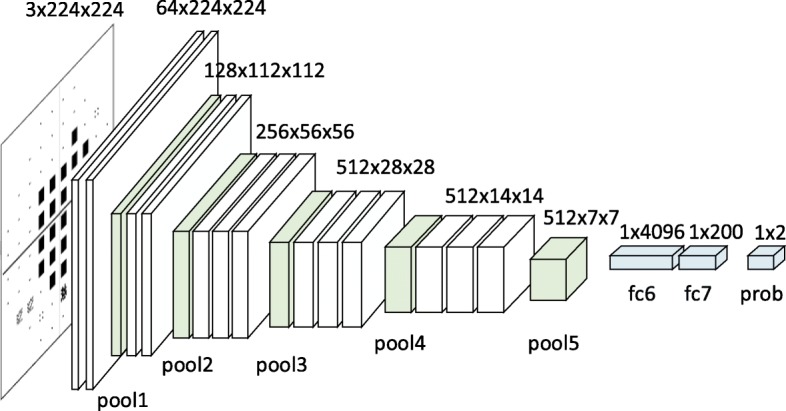
Diagram showing the modified VGG network. VGG15 was adopted as our network structure. We modified the output dimension of the penultimate layer fc7 from 4096 to 200. And the last layer is modified to output a two-dimension vector which corresponds to the prediction scores of healthy VF and glaucoma VF. The network is first pre-trained on a large scale, natural image classification dataset ImageNet16 to initialize its parameters. Then we modified the last two layers as mentioned above and initialized their parameters by drawing from a Gaussian distribution

We adopted the powerful VGG [15, 16] as our network structure. The VGG network consists of 13 convolution layers and 3 fully connected layers. We modified the output dimension of the penultimate layer fc7 from 4096 to 200. And the last layer is modified to output a two-dimension vector which corresponds to the prediction scores of healthy VF and glaucoma VF. The network is first pre-trained on a large scale, natural image classification dataset ImageNet [17] to initialize its parameters. Then we modified the last two layers as mentioned above and initialized their parameters by drawing from a Gaussian distribution. All the parameters of the network were updated by the stochastic gradient descend algorithm with the softmax cross-entropy loss. The network structure is shown in Fig. 1.

### Comparison between CNN-based algorithm and human ophthalmologists in differentiation of VFs

We compared diagnostic accuracy between our algorithm based on deep neural network and ophthalmologists. We chose 9 ophthalmologists in 3 different levels (glaucoma experts: Professor YL-L, XC-D and SJ-F; attending ophthalmologists: Dr. T-S, WY-L and WY-Y; resident ophthalmologists: Dr. X-G, WJ-Z and YY-W), from 4 eye institutes (see details in acknowledgements). None of them has participated in the current research. Attending ophthalmologists are doctors who have clinical training in ophthalmology for at least 5 years, while resident ophthalmologists are doctors who have clinical training in ophthalmology for 1–3 years. Ophthalmologists were shown the PD images alone and requested to assign one of five labels to each PD image, i.e., non-glaucoma, likely non-glaucoma, uncertain, likely glaucoma and glaucoma.

### Traditional methods for VF differentiation

As a comparison, we also evaluated several rule-based methods and traditional machine learning methods for glaucoma diagnosis.

Rule-based methods included AGIS and GSS methods. For AGIS, a VF is considered to be abnormal if three or more contiguous points in the TD plot are outside of normal limits. [9] GSS2 uses both MD and PSD values to classify VFs into 6 stages. [10] Only stage 0 is considered healthy and other stages are treated as glaucoma.

Moreover, we also compared our method with three other non-deep machine learning algorithms. Support Vector Machine (SVM) [18] maps training samples into high dimensional points that can be separated by a hyperplane as wide as possible. Random Forest (RF) [19] constructs a set of decision trees, and each sample is classified according to the number of training samples of different categories falling into the same leaf node. For k-Nearest-Neighbor (k-NN) [20] method, the sample is classified as non-glaucoma or glaucoma by majority voting from its k nearest training samples. Throughout these experiments, we used 52 PD values in VFs obtained in 24–2 test. For 30–2 test, 22 outermost values were discarded so that they can be treated equally. We optimized all the algorithms to improve their performance, e.g., we experimented whether to use Principal Component Analysis (PCA) for preprocessing, different kernel types in SVM, different numbers of trees in RF and various k values in k-NN.

## Results

Baseline characteristics are shown in Table 1. We totally collected 4012 VF reports, including glaucoma and non-glaucoma reports. To compare the statistical difference between non-glaucoma group and glaucoma group, we run an unpaired test for numerical data and chi-square test for categorical data. It can be observed that there was no significant difference between left eye to right eye ratio (*P* = 0.6211, chi-square test), while age (*P* = 0.0022, unpaired t test), VFI (*P* = 0.0001, unpaired t test), MD (*P* = 0.0039, unpaired t test) and PSD (*P* = 0.0001, unpaired t test) exhibited obvious statistical differences.

**Table 1:** Baseline characteristics of participants

	Non-glaucoma Group	Glaucoma Group	*P* Value
No. of images	1623	2389	–
Age (SD)	47.2 (17.4)	49.2 (16.3)	0.0022*
left/right	635/919	607/911	0.6211
VFI (SD)	0.917 (0.126)	0.847 (0.162)	0.0001*
MD (SD)	− 5.0 (23.5)	−9.0 (44.8)	0.0039*
PSD (SD)	3.6 (3.3)	6.7 (22.2)	0.0001*

*shows results with a significant difference

To evaluate the effectiveness of the algorithm for differentiation of glaucoma and non-glaucoma VFs, we summarized the performance of the proposed algorithm in Table 2.

**Table 2:** Performance of the algorithm and the compared methods

	Methods	Accuracy	Specificity	Sensitivity
Ophthalmologists	resident #1	0.640	0.767	0.513
	resident #2	0.593	0.680	0.507
	resident #3	0.587	0.630	0.540
	attending #1	0.533	0.213	0.853
	attending #2	0.570	0.670	0.473
	attending #3	0.653	0.547	0.760
	glaucoma expert #1	0.663	0.700	0.647
	glaucoma expert #2	0.607	0.527	0.687
	glaucoma expert #3	0.607	0.913	0.300
Rule based methods	AGIS	0.459	0.560	0.343
	GSS2	0.523	0.500	0.550
Traditional machine learning methods	SVM	0.670	0.618	0.733
	RF	0.644	0.453	0.863
	k-NN	0.591	0.347	0.870
	CNN	0.876	0.826	0.932

On the validation set of 300 VFs, our algorithm based on CNN achieved an accuracy of 0.876, while the specificity and sensitivity was 0.826 and 0.932, respectively. In order to compare the results of ophthalmologists with machines, we also developed a software to collect evaluation results from ophthalmologists. Ophthalmologists were shown the PD images alone and requested to assign one of five labels to each image, i.e., non-glaucoma, likely non-glaucoma, uncertain, likely glaucoma and glaucoma. They were strongly advised not to choose the uncertain label. For final evaluation, the non-glaucoma and likely non-glaucoma labels were counted as normal, while the likely glaucoma and glaucoma labels were counted as glaucoma, and the uncertain level is considered as a wrong answer. Although the ophthalmologists included three resident ophthalmologists, three attending ophthalmologists and three glaucoma experts, we did not observe significant differences among these three groups. The average accuracies are 0.607, 0.585 and 0.626 for resident ophthalmologists, attending ophthalmologists and glaucoma experts, respectively. However, there exists a huge performance gap between ophthalmologists and CNN, which indicates that CNN may have strong ability to identify the complex patterns presented in the PD images for glaucoma diagnosis. Two rule-based methods, AGIS and GSS2, were also compared in the experiment. Both methods are not able to achieve satisfactory results. Interestingly, all the ophthalmologists performed better than GSS2 and AGIS, indicating the importance of human experience in the decision-making process. Three traditional machine learning algorithms were also included in the experiments. SVM performed best among these machine learning methods, but still much worse than CNN.

**Fig. 2:**
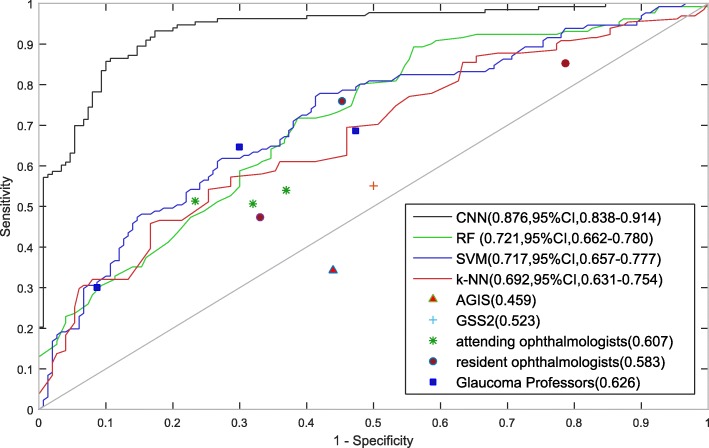
Validation set performance for glaucoma diagnosis. Performance of CNN, ophthalmologists and traditional algorithms are presented. There were 9 ophthalmologists participating in evaluation of VFs. On the validation set of 300 VFs, CNN achieved an accuracy of 0.876, while the specificity and sensitivity was 0.826 and 0.932, respectively. The average accuracies are 0.607, 0.585 and 0.626 for resident ophthalmologists, attending ophthalmologists and glaucoma experts, respectively. Both AGIS and GSS2 are not able to achieve satisfactory results. Three traditional machine learning algorithms were also included in the experiments. SVM performed best among these machine learning methods, but still much worse than CNN. We also examined the receiver operating characteristic curve (ROC) of CNN and the compared methods. CNN achieved an AUC of 0.966 (95%CI, 0.948–0.985), which outperformed all the ophthalmologists, rule based methods and traditional machine learning methods by a large margin

As shown in Fig. 2, we examined the receiver operating characteristic curve (ROC) of CNN and the compared methods. Our algorithm achieved an AUC of 0.966 (95%CI, 0.948–0.985). It outperformed all the ophthalmologists, rule based methods and traditional machine learning methods by a large margin.

**Fig. 3:**
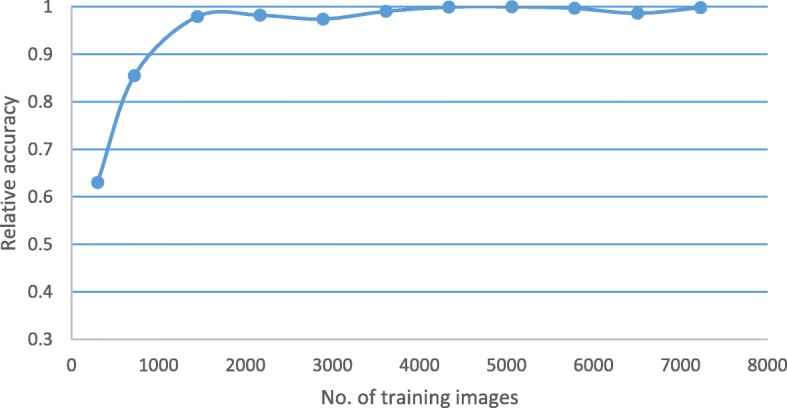
Relative validation set accuracy versus number of training images. We studied the relative validation set accuracy as a function of the number of images in the training set. The training set is randomly chosen as a subset of the original training set at rates of (5%, 10%, …, 100%). Each set includes all the images in the smaller subset. As shown in the figure, the performance does not improve too much after the training set includes more than 3712 images

We also studied the relative validation set accuracy as a function of the number of images in the training set. The training set is randomly chosen as a subset of the original training set at rates of (5%, 10%, …, 100%). Each set includes all the images in the smaller subset. As shown in Fig. 3, we can see the performance does not improve too much after the training set includes more than 3612 images.

## Discussion

In our study, we presented two meaningful contributions: 1) we designed a project to develop our algorithm for differentiation of VFs, which consisted of 4 steps: data collection, model design, training strategy design and model validation; 2) we have developed a deep learning based method that can differentiate glaucoma from non-glaucoma VFs and verified its efficacy on interpretation of VFs and advantage over human ophthalmologists. Our approach based on CNN achieved both higher sensitivity and specificity than traditional machine learning method and the algorithms concluded from clinical trials such as AGIS. [9] Applying CNN to the interpretation of VFs, we found that the method is both sensitive and reliable. Although ophthalmologists performed better than AGIS and GSS2, CNN-based algorithm is even better at recognizing patterns presented in the PD images. Our results demonstrated the possibility of applying CNN to assist screening and diagnosis of glaucoma.

In clinical practice, diagnosis of glaucoma usually needs combination of different test results including VF, OCT and fundus photo. For some glaucoma patients, they may show typical patterns in VF directly indicating glaucomatous damage. [21] However, for other patients, patterns in their VFs are untypical and hard to recognize or interpret. On this occasion, it’s hard to determine whether the VF belongs to a glaucoma patient or not. Because CNN is able to learn from mass data and summarize its own rules of judgement, we designed this research and tried to develop a self-designed algorithm based on CNN to see whether well-trained deep networks have the ability to able to extract effective patterns from VFs for glaucoma discrimination and even find hidden clues that human experts cannot recognize.

In our first step, we compared the performance of our algorithm based on CNN against human ophthalmologists of different levels. As expected, glaucoma experts achieved the highest accuracy in VF interpretation, although there was just 2% and 4% different when compared to attending and resident doctors respectively. With accumulation of clinical experience, doctors tend to have higher specificity while lower sensitivity. Such results are understandable. Because doctors only have VFs as accessory examination to make a diagnosis, their diagnostic ability was restricted, and they would tend to be more careful about their decision. However, machines got the highest score in the test, achieving highest sensitivity while keeping high specificity. In our second step, we compared performance of our algorithm against 2 criteria summarized from clinical trials, AGIS and GSS2. [9, 10] AGIS and GSS2 criteria were built to evaluate severity and staging of glaucoma based on VF. VF is divided into different areas with different weights. These algorithms, however, were based on regression analysis, so it is typically linear and won’t have good performance with complex VFs. In the last step, we compared performance of our CNN-based algorithm with traditional machine learning method, including RF, SVM and k-NN. A previous study used feed forward neural network (FNN) to detect preperimetric glaucoma, which showed overwhelming advantage over traditional machine learning methods. [13] In our study, similar results were obtained. This is because these algorithms are all shallow models which cannot extract representative features of the PD images.

VFs have various patterns, making them complex to interpret. With current techniques, it is still difficult to explain the details on how CNN works to differentiate different VFs. But we are certain that CNN has used a non-linear algorithm by stacking multiple linear and non-linear layers in interpretation of VFs, which is obviously better than humans and other algorithms. When looking at a VF report, ophthalmologists tend to find specific pattern from it. Relationship between adjacent and distant test points may also matter, but there is no theory built about these aspects. If we can extract the patterns or rules used by CNN in differentiation of VFs, it may greatly help clinical diagnosis of glaucoma, which will be our future work.

It should be noted that this study had several limitations. First, we used only pattern deviation images as the input of machine learning algorithms. Thus, preperimetric glaucoma may not be effectively detected by machine. We don’t consider VF from cross-sectional test is able to help diagnose early stage disease, that’s why we didn’t try to differentiate preperimetric glaucoma in our study. In future studies, we plan to combine VF with OCT scans. With input from different imaging modalities, it is expected that deep networks may be able to make more accurate diagnosis. Second, at current stage, the program we developed can just tell glaucoma from non-glaucoma VFs. Various diseases, such as neuro-ophthalmic diseases and cataract, may influence VFs. We hope to extend the function of our deep models to diagnose more ocular diseases.

## Conclusion

In summary, our algorithm based on CNN has achieved higher accuracy compared to human ophthalmologists and traditional rules (AGIS and GSS2). The accuracy is 0.876, while the specificity and sensitivity are 0.826 and 0.932, respectively, indicating advantages of CNN-based algorithms over humans in diagnosis of glaucoma. It will be a powerful tool to distinguish glaucoma from non-glaucoma VFs and may help screening and diagnosis of glaucoma in the future.

## Additional files

Additional file 1: **Figure S1.** Representative examples of pattern deviation figures in glaucomatous and non-glaucomatous visual fields. (TIFF 696 kb)

Additional file 2: Information of affliations of people who contributed to the study but are not in the author list. (DOCX 15 kb)

## Abbreviations

AGIS: Advanced Glaucoma Intervention Study
AI: Artificial intelligence
CC: Congenital cataract
CNN: Convolutional neural network
DR: Diabetic retinopathy
GSS: Glaucoma Staging System
OCT: Optical coherence tomography
ONH: Optic nerve head
RNFL: Retinal nerve fiber layer
VF: Visual field

## References

[1] Quigley HA (2011). Glaucoma. Lancet.

[2] Liang YB, Friedman DS, Zhou Q, Yang X, Sun LP, Guo LX, Tao QS, Chang DS, Wang NL, Handan Eye Study G (2011). Prevalence of primary open angle glaucoma in a rural adult Chinese population: the Handan eye study. Invest Ophthalmol Vis Sci.

[3] Wang YX, Xu L, Yang H, Jonas JB (2010). Prevalence of glaucoma in North China: the Beijing eye study. Am J Ophthalmol.

[4] He M, Foster PJ, Ge J, Huang W, Zheng Y, Friedman DS, Lee PS, Khaw PT (2006). Prevalence and clinical characteristics of glaucoma in adult Chinese: a population-based study in Liwan District, Guangzhou. Invest Ophthalmol Vis Sci.

[5] Jonas JB, Aung T, Bourne RR, Bron AM, Ritch R, Panda-Jonas S. Glaucoma. Lancet. 2017; 10.1016/S0140-6736(17)31469-1 28577860

[6] The Advanced Glaucoma Intervention Study (AGIS) (2000). 7. The relationship between control of intraocular pressure and visual field deterioration.The AGIS Investigators. Am J Ophthalmol.

[7] Musch DC, Gillespie BW, Lichter PR, Niziol LM, Janz NK, Investigators CS (2009). Visual field progression in the collaborative initial Glaucoma treatment study the impact of treatment and other baseline factors. Ophthalmology.

[8] Nouri-Mahdavi K, Hoffman D, Gaasterland D, Caprioli J (2004). Prediction of visual field progression in glaucoma. Invest Ophthalmol Vis Sci.

[9] Advanced Glaucoma Intervention Study. 2 (1994). Visual field test scoring and reliability. Ophthalmology.

[10] Brusini P, Filacorda S (2006). Enhanced Glaucoma staging system (GSS 2) for classifying functional damage in glaucoma. J Glaucoma.

[11] Gulshan V, Peng L, Coram M, Stumpe MC, Wu D, Narayanaswamy A, Venugopalan S, Widner K, Madams T, Cuadros J (2016). Development and validation of a deep learning algorithm for detection of diabetic retinopathy in retinal fundus photographs. JAMA.

[12] Long E, Lin H, Liu Z, Wu X, Wang L, Jiang J, An Y, Lin Z, Li X, Chen J (2017). An artificial intelligence platform for the multihospital collaborative management of congenital cataracts. Nat Biomed Eng.

[13] Asaoka R, Murata H, Iwase A, Araie M (2016). Detecting Preperimetric Glaucoma with standard automated Perimetry using a deep learning classifier. Ophthalmology.

[14] Garway-Heath DF, Crabb DP, Bunce C, Lascaratos G, Amalfitano F, Anand N, Azuara-Blanco A, Bourne RR, Broadway DC, Cunliffe IA (2015). Latanoprost for open-angle glaucoma (UKGTS): a randomised, multicentre, placebo-controlled trial. Lancet.

[15] Chatfield K, Simonyan K, Vedaldi A, Zisserman A (2014). Return of the devil in the details: delving deep into convolutional nets. arXiv preprint arXiv:14053531.

[16] Simonyan K, Zisserman A (2014). Very deep convolutional networks for large-scale image recognition. arXiv preprint arXiv:14091556.

[17] Russakovsky O, Deng J, Su H, Krause J, Satheesh S, Ma S, Huang Z, Karpathy A, Khosla A, Bernstein M (2015). Imagenet large scale visual recognition challenge. Int J Comput Vis.

[18] Cortes C, Vapnik V (1995). Support-vector networks. Mach Learn.

[19] Ho TK (1995). Random decision forests. Document Analysis and Recognition, 1995, Proceedings of the Third International Conference on: 1995: IEEE.

[20] Altman NS (1992). An introduction to kernel and nearest-neighbor nonparametric regression. Am Stat.

[21] Atalay E, Nongpiur ME, Yap SC, Wong TT, Goh D, Husain R, Perera SA, Aung T (2016). Pattern of visual field loss in primary angle-closure Glaucoma across different severity levels. Ophthalmology.

