# Correction to: Automatic differentiation of Glaucoma visual field from non-glaucoma visual field using deep convolutional neural network

**DOI:** 10.1186/s12880-019-0339-z

**Published:** 2019-05-21

**Authors:** Fei Li, Zhe Wang, Guoxiang Qu, Diping Song, Ye Yuan, Yang Xu, Kai Gao, Guangwei Luo, Zegu Xiao, Dennis S. C. Lam, Hua Zhong, Yu Qiao, Xiulan Zhang

**Affiliations:** 1 Zhongshan Ophthalmic Center, State Key Laboratory of Ophthalmology, Sun Yat-sen University, Guangzhou, China; 2 Guangdong key lab of Computer Vision & Virtual Reality, Multimedia Research Center, Shenzhen Institutes of Advanced Technology, Chinese Academy of Sciences, Shenzhen, China; 3 Department of Ophthalmology, The First Affiliated Hospital of Kunming Medical University, Kunming, China; 4 SenseTime Group Limited, Hong Kong, China; 5 C-MER Dennis Lam Eye Hospital, Shenzhen, China

**Correction to: BMC Med Imaging (2018) 18: 35**

**https://doi.org/10.1186/s12880-018-0273-5**

In the original version of this article [1], published on 4 October 2018, there was an error in the article title. The title of the original publication is: *“Automatic differentiation of Glaucoma visual field from non-glaucoma visual* ***filed*** *using deep convolutional neural network*”, however, the correct title should be: “*Automatic differentiation of Glaucoma visual field from non-glaucoma visual* ***field*** *using deep convolutional neural network*”, as presented in the title of this Correction article.

The same error appears in the Background section of the Abstract. *"To develop a deep neural network able to differentiate glaucoma from non-glaucoma visual fields based on visual filed (VF) test results, we collected VF tests from 3 different ophthalmic centers in mainland China"* should instead read: *"To develop a deep neural network able to differentiate glaucoma from non-glaucoma visual fields based on visual field (VF) test results, we collected VF tests from 3 different ophthalmic centers in mainland China"*.
