# Supplementary figures and images for: Automatic differentiation of Glaucoma visual field from non-glaucoma visual filed using deep convolutional neural network
Source: BMC Med Imaging. 2018 Oct 4;18:35. doi: 10.1186/s12880-018-0273-5 (PMC6172715; doi:10.1186/s12880-018-0273-5)

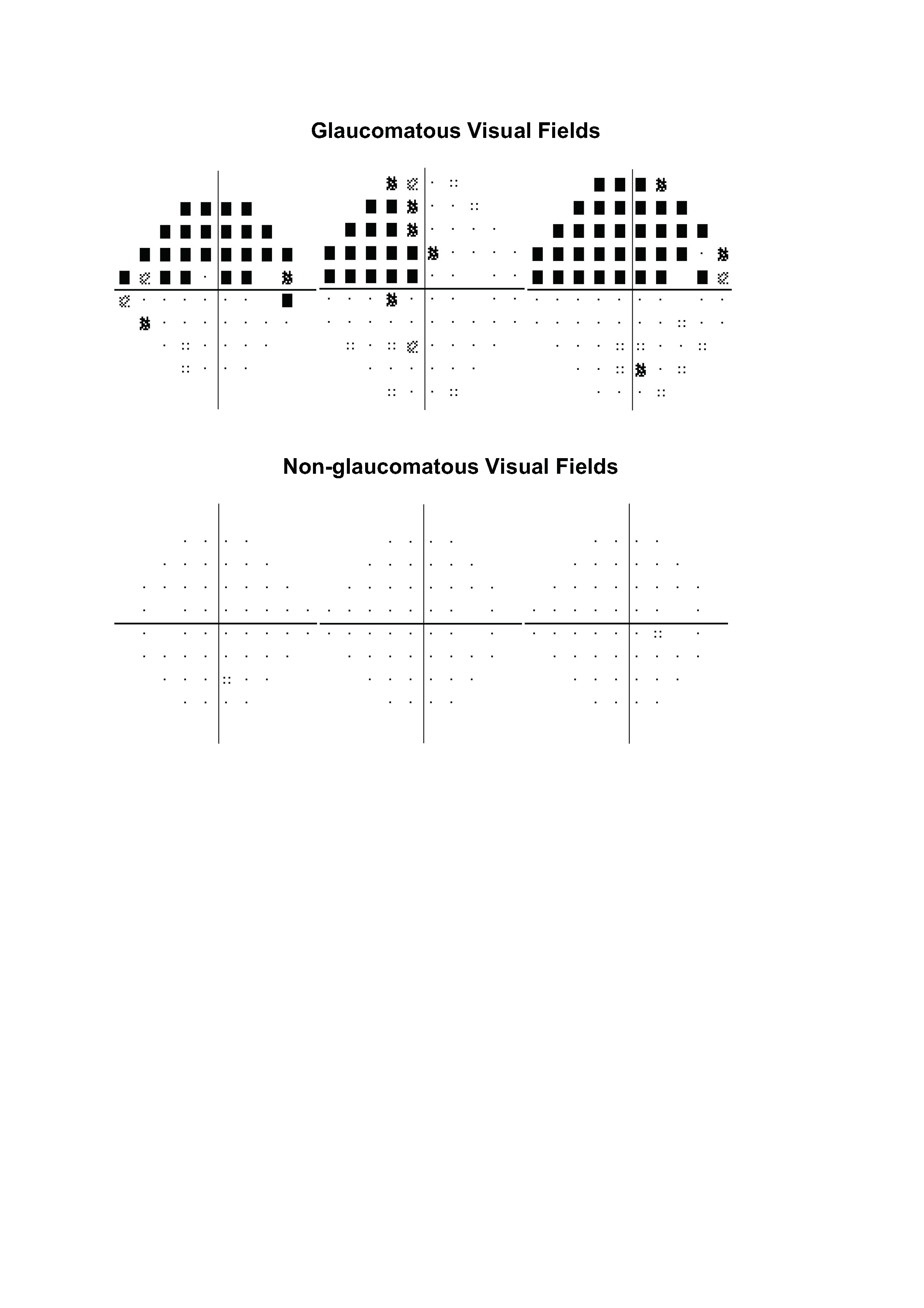

Supplement: Supplementary file 1 — Figure S1. Representative examples of pattern deviation figures in glaucomatous and non-glaucomatous visual fields. (TIFF 696 kb) [file 12880_2018_273_MOESM1_ESM.tiff]
