# Supplementary material for: Automatic differentiation of Glaucoma visual field from non-glaucoma visual filed using deep convolutional neural network
Source: BMC Med Imaging. 2018 Oct 4;18:35. doi: 10.1186/s12880-018-0273-5 (PMC6172715; doi:10.1186/s12880-018-0273-5)
Supplement: Supplementary file 2 — Information of affliations of people who contributed to the study but are not in the author list. (DOCX 15 kb) [file 12880_2018_273_MOESM2_ESM.docx]

**Affliations:**

1. Prof. Yunlan Ling, Prof Guangwei Luo and Dr.Tao Shen are from Zhongshan Ophthalmic Center, Guangzhou, China.
2. Prof. Xuanchu Duan: Department of Ophthalmology Second Xiangya Hospital, Central South University, Changsha, China.
3. Prof. Sujie Fan: Department of Ophthalmology, Handan Eye Hospital, Handan, China.
4. Dr. Wenyan Yang and Yuanyuan Wang: Department of Ophthalmology, the First Affiliated Hospital of Kunming Medical University, Kunming, China.
5. Dr. Weiying Liu and Wenjie Zhang: C-MER Dennis Lam Eye Hospital, Shenzhen, China
6. Dr. Xin Guo: Department of Ophthalmology, the Second Affiliated Hospital of Guangzhou Medical University, Guangzhou, China.
7. Prof. Hongsheng Li: Department of Electronic Eningeering, the Chinese University of Hong Kong, Hong Kong, China
